# Microfluidic Characterization and Analysis of Circulating Tumor Cells From Patients With Metastatic Melanoma

**DOI:** 10.1111/pcmr.70030

**Published:** 2025-06-02

**Authors:** Matthew C. Mannino, Shuang G. Zhao, Benjamin K. Gibbs, Jennifer L. Schehr, Isabella G. Fernandez, Diego A. Eyzaguirre, Alyssa M. Hintz, Stephanie J. Davis, Manushi N. Vatani, Jacob C. Caceres, Alexander Birbrair, Joshua M. Lang, Vincent T. Ma

**Affiliations:** ^1^ University of Wisconsin‐Madison Madison Wisconsin USA; ^2^ Department of Human Oncology University of Wisconsin‐Madison Madison Wisconsin USA; ^3^ Department of Dermatology University of Wisconsin‐Madison Madison Wisconsin USA; ^4^ Division of Hematology, Department of Medicine, Medical Oncology, and Palliative Care University of Wisconsin‐Madison Madison Wisconsin USA

**Keywords:** circulating tumor cell, fluorescence microscopy, human leukocyte antigen class I, immune checkpoint inhibitor, liquid biopsy, melanoma, programmed death Ligand‐1

## Abstract

Circulating tumor cells (CTCs) can provide non‐invasive insight into how a cancer patient responds to therapy. Their role in disease monitoring of advanced melanoma patients treated with immune checkpoint inhibitors (ICI) is unknown. CTC protein expression of human leukocyte antigen class‐I (HLA I) and programmed death ligand‐1 (PD‐L1) may give insight into how a patient's disease evolves over the course of treatment. In our study, we utilize microfluidic Exclusion‐based Sample Preparation (ESP) technology to isolate and characterize CTCs from patients with advanced‐stage melanoma. CTC samples from melanoma patients are collected, captured, and stained. A range of 2 to 35 CTCs is observed in a cohort of 16 samples from 10 advanced‐stage melanoma patients treated with ICI therapy. Single‐cell protein expression data is generated from image cytometry analysis and used to calculate mean HLA I and PD‐L1 expression. Using our ESP capture approach, we successfully detect phenotypic and numerical heterogeneity in CTCs from melanoma patients. Our assay shows sufficient capture sensitivity and promising prognostic and predictive information, as we illustrate in our case example. A greater clinical sample size will be necessary to confirm the diagnostic sensitivity and specificity of the assay in predicting clinical outcomes for patients with advanced‐stage melanoma.

## Graphical abstract

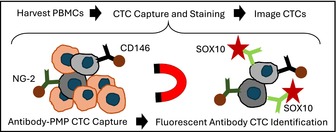

**Summary**

- We present a robust protocol for isolating and histologically characterizing circulating tumor cells.
- Using our protocol, we find that circulating tumor cells can be potentially used for longitudinal disease tracking.
- The multi‐target capture strategy is tested on multiple melanoma cell lines.
- The complete CTC capture and characterization protocol is preliminarily tested on 10 patients with advanced‐stage melanoma.
- One of the patient's CTC and ctDNA levels are tracked longitudinally alongside concurrent radiographic assessments to confirm the protocol's preliminary potential for clinical correlation.

## 1 Introduction

Immune checkpoint inhibitors (ICIs) can result in robust and durable disease control in many advanced cancers, and their approval continues to expand rapidly. Melanoma has been at the forefront of ICI advancements over the past decade (Knight et al. 2023). Despite the benefit that these treatments give some patients, other patients can still progress with tumor cells evolving to use alternate mechanisms of immune evasion. Example immune regulatory protein targets of interest in the field of melanoma include programmed cell death protein 1 (PD‐1), programmed death ligand‐1 (PD‐L1), cytotoxic T‐cell lymphocyte antigen‐4 (CTLA‐4), and lymphocyte‐activation gene 3 (LAG‐3). Molecular machinery related to these signaling pathways may also provide insight; for example, functional cancer‐associated antigen presentation through human leukocyte antigen class I (HLA I) (Sabbatino et al. 2020).

Due to the constant evolution and progression melanoma undergoes, readily available methods of disease evaluation are necessary for optimizing patient care and outcomes. Currently, no biomarkers are used in clinical practice to help inform therapy selection for melanoma patients. The most heavily investigated biomarker for ICI therapy is the expression of PD‐L1 on tumor cells and tumor‐infiltrating lymphocytes (Akiyama et al. 2020). Studies have demonstrated mixed results correlating PD‐L1 expression with clinical efficacy to ICI in advanced melanoma (Morrison et al. 2018; Oh et al. 2021). Other biomarkers that have been explored include tumor mutational burden (TMB), TILs, and RNA gene expression signatures, with variable results such that no single biomarker has been proven to be an effective predictor of ICI‐based treatment outcomes in melanoma (Ding et al. 2022). This suggests the need for a comprehensive real‐time assay that can capture the interplay between multiple integrated molecular and immunological components that each have differing but important roles in response outcome.

The lack of clinically available biomarkers stems, in part, from limitations in existing biomarker studies, including limitations of tissue availability on trials, difficulty procuring real‐time tumor biopsies, and use of isolated metastatic sites. Because of this, these studies do not fully capture the scope of tumor heterogeneity or necessarily capture the dominant/drug‐resistant clones. Technologies for non‐invasive “liquid” biopsies exist that can overcome many limitations of tissue‐based biopsies, using blood draws from patients to isolate biomarkers such as circulating tumor cells (CTCs) or circulating tumor DNA (ctDNA) and have shown prognostic potential in various different cancers, including melanoma (Khoja et al. 2013; Santiago‐Walker et al. 2016; Dell'Oro et al. 2024; Egger et al. 2024; Braune et al. 2020; Khaddour et al. 2022; Brunsgaard et al. 2023; Eroglu et al. 2023; Ma et al. 2024). Clinical implementation of existing melanoma liquid biopsy methods has been delayed by problems with analytical and clinical sensitivity, specificity, and ease of use (Aya‐Bonilla et al. 2017; Freeman et al. 2012; Khoja et al. 2013; Tivey et al. 2022). The S100 antigen, for example, has been used as a determining criterion for diagnosing melanoma in sentinel lymph node biopsies (SLNB), but low specificity makes it an inferior candidate for use in liquid biopsies.

SOX10 has previously been identified as a higher‐specificity biomarker in recent melanoma literature (Szumera‐Ciećkiewicz et al. 2020; Willis et al. 2015) and was therefore selected as the melanoma‐specific CTC identifier in this study. Microfluidic Exclusion‐based Sample Preparation (ESP) technology is used here to enable automated isolation of CTCs combined with quantitative image cytometry for high precision CTC enumeration and biomarker evaluation (Schehr et al. 2022). Similar to others, CD146, also referred to as melanoma cell adhesion molecule (MCAM), is used to capture melanoma CTCs (Rao et al. 2011; Freeman et al. 2012; Aya‐Bonilla et al. 2017; Beasley et al. 2021; Kang et al. 2022), as this marker has been described as being nearly universally expressed in melanoma (Medic et al. 2007). Given other reports demonstrating overexpression and capture capability of Neuron‐Glial Antigen‐2 (NG2), also referred to as Melanoma‐associated Chondroitin Sulfate Proteoglycan (MCSP), in melanoma and several different treatment‐resistant cancers (Li et al. 2003; Beasley et al. 2021; Ulmer et al. 2008, 2004; Suesskind et al. 2009; Kitago et al. 2009; Sakaizawa et al. 2012; Kang et al. 2022), a multi‐target capture approach is utilized for a more comprehensive understanding of the disease. Here, we demonstrate the preliminary analytical and clinical promise of this microfluidic ESP‐based melanoma CTC characterization approach. See Table 1 for the materials and equipment list.

**TABLE 1:** Materials and equipment list.

Resource	Purpose/function	Vendor/manufacturer
RPMI‐1640	Cell culture maintenance	Corning—#MT10040CV
Fetal Bovine Serum	Cell culture Maintenance/staining	Life Technologies—#10438026
Penicillin/Streptomycin	Cell culture maintenance	VWR—#16777‐164
Melanoma Patient Blood	Patient blood collection	UWCCC IRB #2014‐1214
Ficoll‐Paque	Blood processing	Cytiva—# 45‐001‐750
VitaStain Acridine Orange Propidium Iodide (AOPI) Solution	Blood processing	Revvity—#CS2‐0106‐5ML
Cellometer Auto2000 Cell Viability Counter	Blood processing	Revvity—#CMT‐A2K
LS Column	Blood processing	Miltenyi—#130‐042‐401
CD45 MACS Beads	Blood processing	Miltenyi—#130‐045‐801
MACS Buffer	Blood processing	Miltenyi—#130‐091‐221
Tween‐20	Cell line/CTC capture	ThermoFisher—#BP337‐500
1X PBS	Cell line/CTC capture	ThermoFisher—#10010072
Anti‐NG2 Antibody	Cell line/CTC capture	Invitrogen—#14‐6504‐82
Anti‐CD146 Antibody	Cell line/CTC capture	BioLegend—#361036
SeraMag Beads	Cell line/CTC capture	VWR—#10204‐622
Robot Capture Strips	Cell line/CTC capture	Gilson—#22100007
Robot ExtractMan Plates	Cell line/CTC capture	Gilson—# 22100018
Antibody DSB‐X Biotinylation Kit	Antibody Biotinylation	Invitrogen—#D20655
EDTA Vacutainer Tubes	Patient Blood Collection	BD—#366643
Hoechst 33342	CTC staining	ThermoFisher—#62249
Anti‐CD45 Antibody	CTC staining	BioLegend—#304017
Anti‐CD34 Antibody	CTC staining	BioLegend—#343518
Anti‐CD66b Antibody	CTC staining	BioLegend—#305104
Anti‐PD‐L1‐APC Antibody	CTC staining	Invitrogen—#17‐5983‐42
Anti‐HLA‐I PE Antibody	CTC staining	BioLegend—#311406
Anti‐SOX10 Antibody	CTC staining	BioLegend—#847202
Antibody AF790 Conjugation Kit	Antibody fluorophore conjugation	Invitrogen—#A20189
Gilson ExtractMax Modified Pipetting Robot	Cell line/CTC capture and staining	Gilson—Custom
Gilson ExtractMan	Cell line/CTC capture and staining	Gilson—#22100000
Nikon Eclipse TI‐2 Fluorescent Microscope	Cell line/CTC microscopy	Nikon
NIS Elements AR v4.51	Image analysis	Nikon

*Note:* Table description of the materials, reagents, and equipment used in sample processing. The table describes the function and vendor information of the necessary resources.

## 2 Materials and Methods

### 2.1 Cell Lines and Antibodies

M21, SK‐Mel‐28, and Mel624 are cultured and maintained in Corning Cellgro RPMI‐1640 (Corning, MT10040CV), with 10% Fetal Bovine Serum (FBS) (Life Technologies, 10438026) and 1% Penicillin/Streptomycin (VWR, 16777‐164). Mel624 cells express PD‐L1 (624‐B7H1) through transfection. Mel624 and Mel624‐B7H1 cell lines are a generous gift from Drew M. Pardoll at Johns Hopkins University, originating from the transfection described previously (Dong et al. 2002). Antibodies against neuron‐glial antigen‐2 [NG2, melanoma‐associated chondroitin sulfate proteoglycan] (Invitrogen, 14‐6504‐82) and CD146 [melanoma cell adhesion molecule (MCAM)] (BioLegend, 361036) are used to capture cell lines and CTCs from patients with advanced stage melanoma. Anti‐NG2 antibody is biotinylated using a commercial biotinylation kit (Invitrogen, D20655).

### 2.2 Patient Sample and Blood Processing

**FIGURE 1:**
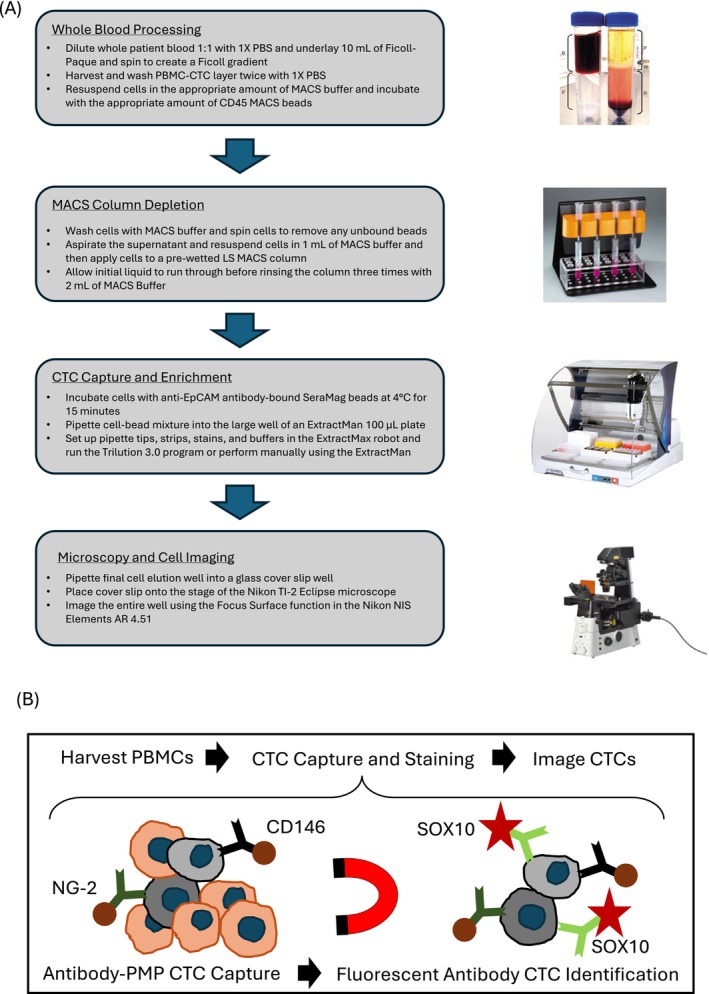
Methodological Schematic. Schematic of blood processing and CTC characterization workflow. (A) PBMCs and CTCs are isolated from whole patient blood by creating a Ficoll gradient (buffy coat). The PBMCs and CTCs are run through a magnetic separation column to remove white blood cells (WBCs). The CTCs are further enriched and then fluorescently stained using a modified Gilson ExtractMax robot. The stained cells are then imaged using fluorescent microscopy and characterized using image analysis software. (B) Antibody‐bound Paramagnetic Particles (PMPs) facilitate the enrichment and subsequent analysis of patient CTCs.

Sixteen whole‐blood samples from 10 patients are collected and processed under IRB approval from the University of Wisconsin‐Madison Carbone Cancer Center (UWCCC) (2014‐1214). All patients provide written consent before enrollment and subsequent blood draws. Detailed patient clinical information is listed in the (Table S1). All patients were anticipated to start systemic treatment at the time of the first blood draw and were serially drawn during the course of therapy. Healthy donors serve as controls and are obtained from the UWCCC BioBank. For blood draws, 20 mL of blood is drawn via venipuncture into 10 mL ethylenediaminetetraacetic acid (EDTA) Vacutainer tubes. Blood processing is performed (Figure 1) as previously described (Schehr et al. 2022).

### 2.3 CTC and Cell Line Capture

CTCs and cell lines are isolated using ESP technology using the Gilson ExtractMax modified pipetting robot. Cell lines are captured using antibodies against either NG2, CD146, or both. Patient CTCs are captured using antibodies against NG2 and CD146. Capture antibodies are bound to SeraMag beads (VWR, 10204‐622).

### 2.4 Staining and Fluorescence Microscopy

Cell lines are stained using Calcein‐AM. Patient CTCs are stained with Hoechst and fluorescently labeled antibodies against CD45 (BioLegend, 304017), CD34 (BioLegend, 343518), CD66b (BioLegend, 305104), PD‐L1 (Invitrogen, 17‐5983‐42), and HLA‐I (BioLegend, 311406). Cells are then fixed and permeabilized using Invitrogen Foxp3 Transcription Factor Staining Buffer Kit (Invitrogen, 00‐5523‐00), then intracellularly stained with SOX10 (BioLegend, 847202) conjugated to AlexaFluor‐790 (Invitrogen, A20189). Images are taken on a Nikon Eclipse TI‐2 fluorescent microscope (Nikon, USA) as previously described (Schehr et al. 2022).

### 2.5 Image Analysis and Biomarker Expression Evaluation

Image analysis is done using NIS Elements AR v4.51, using background subtraction, rolling ball size 30 or 10px for cell lines or patient samples, respectively. Spot detection is used to automatically identify cells within the large image, followed by manual review to remove non‐cellular artifacts. Cells that are Hoechst+/SOX10+/Exclusion (CD45/34/66b)‐ are determined to be CTCs. Mean Fluorescence Intensity (MFI) is calculated for HLA I and PD‐L1 on CTCs.

### 2.6 Statistical Analysis

Data is exported from NIS Elements into Microsoft Excel, then the MFI of the exclusion and SOX10 channels for each cell are graphed on xy graphs with log scale axes in GraphPad Prism (GraphPad Software, San Diego, CA) to visualize population clustering as described previously (Schehr et al. 2022). Cutoffs for positive antibody staining are defined based on clustering characteristics using the principles of flow cytometry (Shapiro 2003). Statistical calculations are then performed on log‐transformed MFI values, given the log‐normal distribution of fluorescence data (Diwakar 2017). Significance is determined with students' *T*‐tests, considering *p* < 0.05 to be significant.

### 2.7 Protocol for Circulating Tumor Cell Capture and Analysis

#### 2.7.1 Whole Blood Processing

1. Pipette mix whole patient blood using a 10 mL serological pipette
2. Transfer 15 mL of blood from EDTA Vacutainer tube to a 50 mL conical tube
3. Perform a 1:1 dilution by adding 15 mL of 1X PBS to the 50 mL conical tube and pipette mix.
4. Underlay 10 mL of Ficoll‐Paque beneath the diluted blood
5. Spin the tube at 1000 g for 20 min with slow acceleration and deceleration.
6. Transfer the peripheral blood mononuclear cell (PBMC) layer to a new 50 mL conical tube
7. Fill the new conical tube up to the 50 mL mark with 1X PBS
8. Close the tube and invert 2–3 times
9. Transfer 10 μL of this solution to a 96 well plate to count
10. Spin at 300 g for 5 min
11. Count cells during this spin by mixing 10 μL previously removed with 10 μL of Acridine Orange and Propidium Iodide (AOPI) stain.
12. Transfer all 20 μL of the mixture into a Cellometer slide
13. Calculate the total amount of cells based on the concentration given by the Cellometer.
14. Aspirate and discard the supernatant
15. Resuspend the pellet in 50 mL of 1X PBS
16. Spin at 300 g for 5 min
17. Calculate the appropriate volume of CD45 MACS beads and MACS buffer according to the manufacturer protocol.
18. Aspirate and discard the supernatant
19. Resuspend the pellet in the calculated volume of MACS buffer
20. Add the calculated volume of CD45 MACS beads and pipette mix
21. Incubate the cells and beads at 4°C for 15 min
22. Prepare an LS column per manufacturer's protocol by washing with 3 mL of MACS buffer
23. Bring the volume in the conical tube containing cells and beads up to 50 mL with 1x PBS.
24. Spin at 300 g for 5 min
25. Aspirate and discard the supernatant
26. Resuspend the pellet in 1 mL of MACS buffer
27. Transfer to the LS column and allow to flow through into a 15 mL conical tube
28. Rinse the column with 2 mL of MACS buffer and repeat for a total of three rinses.
29. Spin the 15 mL conical tube at 300 g for 5 min
30. Aspirate the supernatant

#### 2.7.2 Antibody‐Bead Preparation

1. Wash 5 μL Sera‐Mag speed beads three times with 25 μL of 1X PBS + 0.1% Tween.
2. Resuspend beads in 25 μL of 1X PBS + 0.1% Tween
3. Add 2 μL of biotinylated anti‐CD146 and 2 μL of biotinylated anti‐NG‐2 antibodies to the beads
4. Place on shaker for 20 min at room temperature
5. Wash the beads three times with 25 μL 10% FBS
6. Resuspend beads in 25 μL 10% FBS

#### 2.7.3 CTC Enrichment and Staining

1. Combine the CD45‐depleted fraction with the antibody‐conjugated SeraMag beads.
2. Bring the volume up to 500 μL with 10% FBS
3. Rotate cells with beads in a 1.5 mL tube for 20 min at 4°C
4. Quick spin down the sample tube and transfer to the large input well of the extractman plate.
5. Add pipette tips, strips, an extractman plate, and reagent tubes to either a Gilson ExtractMax robot or a manual Gilson ExtractMan device.
6. Either run the custom pipetting robot program within the pipetting robot software, Trilution 3.0 Micro, or use the manual Gilson ExtractMan device.

#### 2.7.4 Microscopy Preparation and Imaging

1. Adhere an adhesive‐backed silicone isolator to a glass coverslip
2. Transfer the prepared sample to a single well in the isolator
3. Acquire an image of the entire isolator well using a TI2‐Eclipse microscope with NIS Elements version AR 4.51
4. Turn on Dichroic and Perfect Focus System (PFS) and set an optimal focus offset.
5. Define the focus surface with nine points distributed across the imaging area.
6. Turn off the PFS and Dichroic
7. Acquire the image using the large image acquisition feature, acquiring an 8 × 8 grid of small tiles at 10× magnification, then stitching together to generate a large image.

#### 2.7.5 Image Analysis

1. Refer to (Schehr et al. 2022) for detailed methods on analysis.

### 2.8 Troubleshooting

- PBMCs not separating cleanly from RBCs during the ficoll separation step.
  ○ Ensure a swing‐bucket rotor is used for the centrifugation
  ○ Ensure the blood sample is collected in EDTA tubes and processed within 24 h.
  ○ Ensure the blood is not allowed to warm to 37°C or freeze prior to processing
- Tiles not in‐focus during imaging
  ○ When setting focus surface points, make sure to have the brightfield channel in view while setting the points to ensure focus is maintained when nearing the isolator edge.
  ○ Use a fluorescence microscope setup of an equivalent version as described here, as older versions will not allow focus surface setup while viewing the brightfield channel.
- Difficulty retaining cells if cell yield is low after ficoll or MACS
  ○ If cell number is low (e.g., below 1 × 10^6^ cells per buffy coat), aspirate the supernatant from each of the washes down to just above the bottom of the tube to avoid disturbing or inadvertently aspirating and discarding the cell pellet.

## 3 Pre‐Clinical Example Outcomes

### 3.1 Optimizing Capture Efficiency Using a Multi‐Protein Targeting Strategy

**FIGURE 2:**
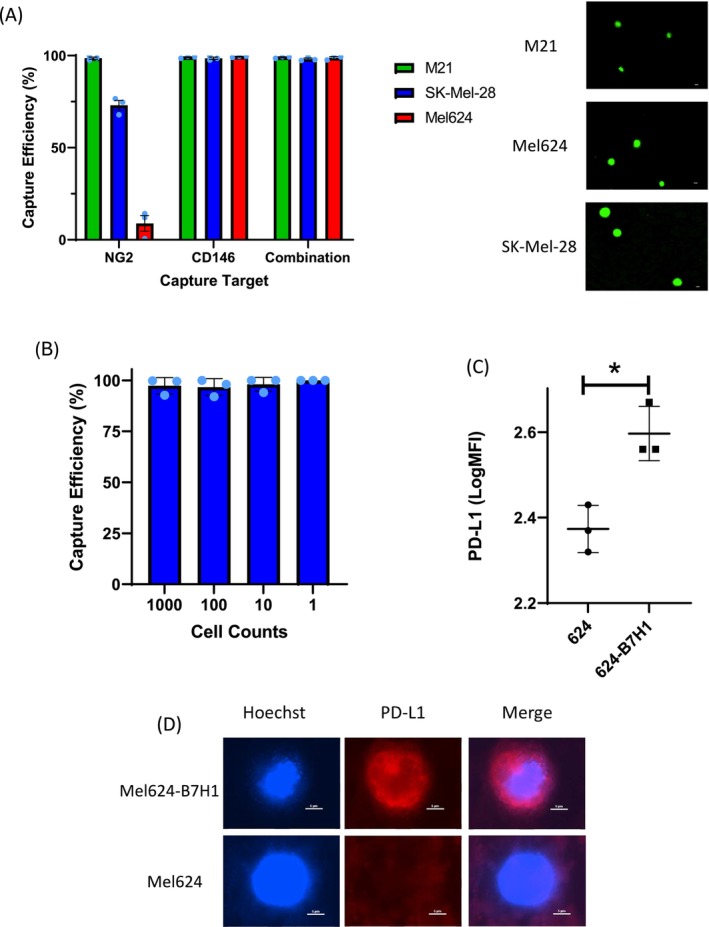
Capture and Staining Validation via Cell Lines. Validation of capture and staining strategy using melanoma cell lines. (A) M21, SK‐Mel‐28, and Mel624 cells were captured using three different protein targeting strategies: NG2, CD146, and both. Representative images shown are 10×. All scale bars shown are 10 μm. Each dot represents the capture efficiency of a single replicate with a total of three replicates run on three separate days. (B) Mel624 cells were captured at serially diluted cell counts ranging from 1000 cells to 1 cell. Each dot represents the capture efficiency of a single replicate with a total of four replicates run on three separate days. (C) Mel624 cells were transfected to express PD‐L1. (D) Both transfected (Mel624‐B7H1) and untransfected Mel624 cells were fluorescently stained for PD‐L1 side‐by‐side. Each symbol represents the average logMFI of all cells from each condition from each of three separate experiments performed on three separate days. Representative images shown are 20×. *Statistically significant at *p* < 0.05.

To confirm that capturing with NG2, in addition to CD146, would not impair CD146 single antibody capture efficiency, we compare capturing with one versus both antibodies. This multi‐target capture strategy is tested on three different melanoma cell lines: Mel624, M21, and SK‐Mel‐28. Multi‐target capture does not impair capture efficiency of either Mel624 or SK‐Mel‐28 cells, when compared to single‐antibody capture. The average capture efficiency using multi‐protein targeting is 99%, compared to 60% or 99% with either NG2 or CD146 alone. (Figure 2A) Once the targeting strategy is validated, the consistency across different cell inputs is tested. The accuracy of this capture approach is demonstrated on a range of 1–1000 Mel624 cells, with at least 95% capture efficiency at each cell count (Figure 2B).

### 3.2 Melanoma Biomarker Staining Specificity

In order to verify the specificity of PD‐L1 staining, PD‐L1 staining is performed on Mel624 cells and Mel624 cells transfected to express PD‐L1 (Mel624‐B7H1). Mel624‐B7H1 cells consistently display significantly higher MFI than the untransfected Mel624 cells (*p* < 0.05). The logMFI of the Mel624 and Mel624‐B7H1 cells is 2.4 and 2.6, respectively. These numbers translate to raw MFIs of 237 and 398, respectively (Figure 2C, Figure 2D). This demonstrates the ability of the technology to reliably differentiate between cells expressing high or low quantities of biomarkers.

## 4 Clinical Correlate Example Outcomes

### 4.1 CTC Identification Marker Selection and Patient Biomarker Heterogeneity

**FIGURE 3:**
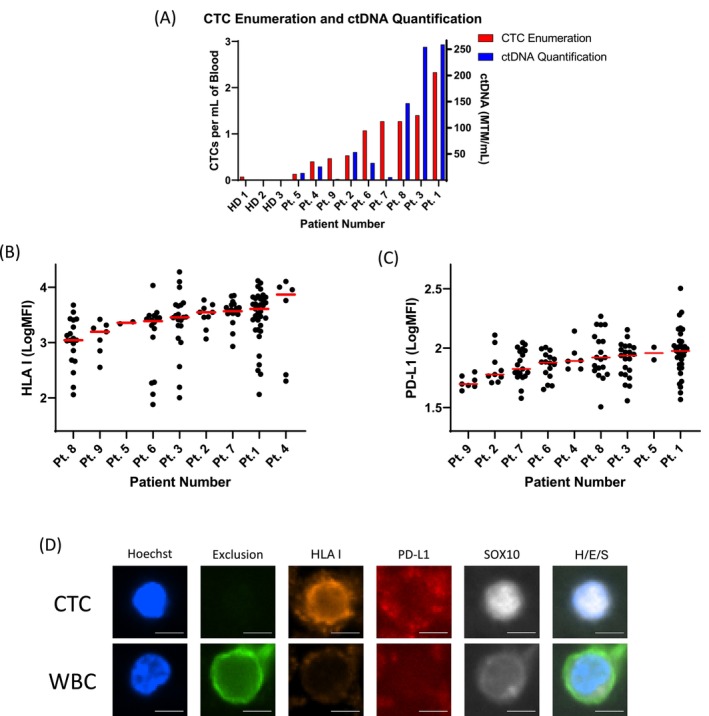
Isolation and Phenotypic Characterization of Patient CTCs. Enumeration and biomarker expression evaluation of Patient CTCs. (A) CTCs were isolated and enumerated from nine total samples from nine total patients. ctDNA (Signatera) was also isolated from patient plasma for seven out of nine of these samples. HD: Healthy Donor. (B) HLA I expression on CTCs was evaluated by fluorescence staining and microscopy. (C) PD‐L1 expression by Patient CTCs was also evaluated by fluorescence staining and microscopy. (D) Representative 20× images of Patient CTCs and White Blood Cells (WBCs) are shown. Merged images include Hoechst, Exclusion, and SOX10 (H/E/S). All scale bars shown are 5 μm.

Total CTC counts and biomarker expression levels are shown for 9 patients with metastatic melanoma; CTC counts range from 2 to 35, with a mean of 15 CTCs (Figure 3A). Correlation with ctDNA (Signatera) levels suggests diagnostic specificity of the CTC results (Figure 3A). LogMFI of HLA‐I on CTCs ranges from 1.88 to 4.28, respectively, with a mean of 3.33 (Figure 3B). LogMFI of PD‐L1 on CTCs ranges from 1.51 to 2.50, with a mean of 1.90 (Figure 3C). The wide range of protein expression levels detected within and between patient sample CTCs suggests the assay may be capable of detecting biological heterogeneity and potentially sensitivity to therapeutic intervention. Three healthy donor controls demonstrate assay specificity (Figure 3A).

### 4.2 Longitudinal Tracking Demonstrates Correlative Potential

**FIGURE 4:**
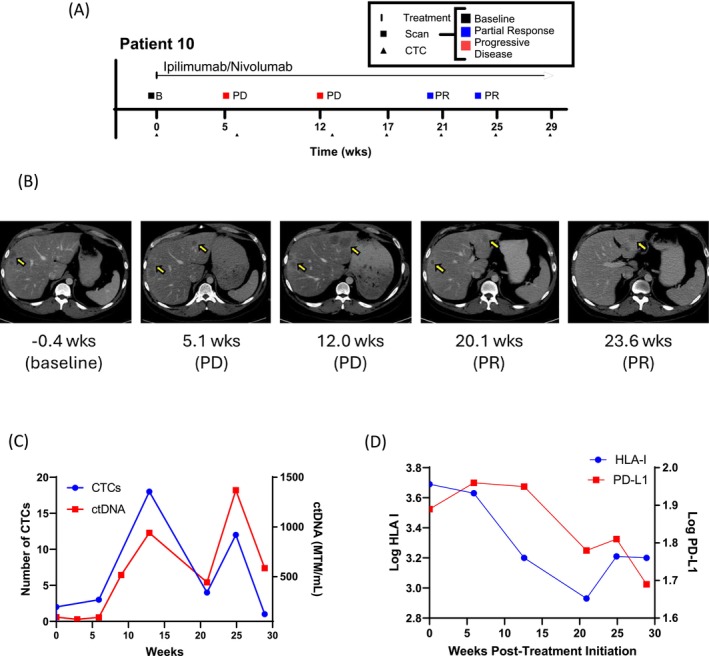
Longitudinal Case Report. Serial radiographic assessments and CTC measurements from a single patient with metastatic melanoma treated with ipilimumab/nivolumab. (A) Course of treatment and tumor response patterns were plotted to illustrate each timeline. (B) Serial CT scans were taken of Patient 10 around each of the respective blood draw timepoints. Notable metastatic liver lesions (labeled with yellow arrows) were followed. (C) CTCs and ctDNA levels were quantified and analyzed longitudinally during the course of treatment. (D) PD‐L1 and HLA I expression levels were tracked during the course of treatment via fluorescence staining and imaging.

A single patient with metastatic (stage IV) melanoma treated with combination ipilimumab (anti‐CTLA‐4) and nivolumab (anti‐PD‐1) had serial blood draws prior to and during the course of treatment. CTCs were enumerated and analyzed from each timepoint. Corresponding radiographic CT scans were performed throughout the course of therapy to evaluate treatment response (Figure 4A). Interpretation of treatment response was determined using RECIST v1.1 (Eisenhauer et al. 2009) by retrospective review. Metastatic liver lesions (labeled with yellow arrows) were followed serially. The patient started treatment with ipilimumab/nivolumab (combination ICI) at week 0. He developed radiographic progressive disease (PD) by week 12 (end of ipilimumab/nivolumab induction). At that point, palliative radiotherapy to his rapidly enlarging cervical lymph nodes was administered concurrently with nivolumab monotherapy. Due to a suspected abscopal effect, he displayed radiographic response (PR) in his liver approximately 20 weeks after the initiation of therapy (Figure 4B). CTC counts showed notable correlation with the patient's treatment response status. All but one of the increases in CTC counts occurred during progressing timepoints. Significant drops in CTC counts occurred during the patient's responding timepoints (Figure 4C) Circulating tumor DNA (ctDNA) was concurrently measured and quantified by using a personalized, tumor‐informed ctDNA assay (Signatera) during the course of the patient's therapy. The fluctuations in the total amount of ctDNA followed a strikingly similar trend to that of the CTC enumeration.

The HLA‐I and PD‐L1 staining intensities were tracked during the course of therapy (Figure 4D). Average CTC PD‐L1 expression remained high initially, but as CTC number dropped and clinical response was observed, PD‐L1 decreased concordantly. Average CTC HLA‐I expression also peaked at the time of the patient's first clinical response. While it is unclear whether these trends are related to the selective pressure of the therapy, these findings warrant further investigation into the clinical value of this biomarker assay.

## 5 Discussion

This study demonstrates the feasibility of an ESP‐based method for capturing and characterizing therapeutic biomarkers on melanoma CTCs. The capture target methodology, capture accuracy, and biomarker staining specificity were tested using multiple cell lines. Patient CTCs were isolated from whole blood draws and stained for identification and characterization, yielding between 2 and 35 CTCs, with an average of 12 CTCs. A total of 6 blood draws and 8 ctDNA samples from a single patient were evaluated to compare concordance with the patient's disease status during systemic therapy. The ctDNA levels correlated well with the CTC counts throughout the course of treatment. Microfluidic ESP technology combined with fluorescence microscopy has enabled effective isolation and phenotypic evaluation of CTCs in other clinical settings (Bootsma et al. 2022; Schehr et al. 2022; Zhao et al. 2022), and our study shows the feasibility of this approach in the melanoma disease setting. This study also illustrates the preliminary clinical value of assessing the dynamic change of CTC enumeration and CTC PD‐L1 and HLA I expression in the advanced melanoma setting during the course of ICI therapy.

Further clinical investigation with this assay is warranted based on this data, with immediate future directions centered around the continual testing of the assay on a larger sample and patient cohort. This study illustrates the ability to capture CTCs in patients across different melanoma subtypes, including cutaneous, mucosal, and uveal melanoma (Table S1). Expanding the study to include greater collection and analysis of bioinformatics from a larger cohort could help confirm the potential clinical utility of the assay. The inclusion of this assay in a prospective clinical trial in melanoma patients could elucidate the utility that the assay might have in detecting or predicting treatment resistance in patients, as well as a greater understanding of tumor evolution and mechanisms of treatment resistance.

The heterogeneity of metastatic melanoma and melanoma CTCs increases the necessity for efficient isolation and accurate characterization in prognostic assays. Various methods and assays have been reported over the years with a wide range of detection (Aya‐Bonilla et al. 2017; Chudziak et al. 2016; Freeman et al. 2012; Khoja et al. 2013). Some of these assays have been evaluated for their prognostic or predictive potential (Khoja et al. 2013). Liquid biopsies are more readily available while avoiding the invasiveness of traditional biopsies and promise to detect and elucidate resistance mechanisms more rapidly. CTC‐based assays could potentially guide treatment choices in order to optimize patient care before or during the treatment trajectory, ultimately extending the lives of patients with cancer.

## Conflicts of Interest

V.T.M. serves as a consultant/advisory board member for Bristol Myers Squibb, Immunocore, Regeneron, Incyte, Replimune, Teiko.Bio, Partner Therapeutics, and Delcath. S.G.Z. is an inventor on unrelated patents licensed to Veracyte, and a family member is an employee of Artera and holds stock in Exact Sciences. M.C.M., B.K.G., J.L.S., I.G.F., D.A.E., A.M.H., S.J.D., M.N.V., J.C.C., and J.M.L. declares no conflicts of interest.

## Supporting information


**Figure S1.** Non‐Melanoma Cell Line Capture Using The Melanoma Assay.

**Table S1.** Patient characteristics.

## Data Availability

The data that support the findings of this study are available from the corresponding author upon reasonable request.
