# Supplementary material for: Microfluidic Characterization and Analysis of Circulating Tumor Cells From Patients With Metastatic Melanoma
Source: Pigment Cell Melanoma Res. 2025 Jun 2;38(4):e70030. doi: 10.1111/pcmr.70030 (PMC12130673; doi:10.1111/pcmr.70030)
Supplement: Supplementary file 1 — Figure S1. Non‐Melanoma Cell Line Capture Using The Melanoma Assay. [file PCMR-38-0-s001.pptx]

## Slide 1
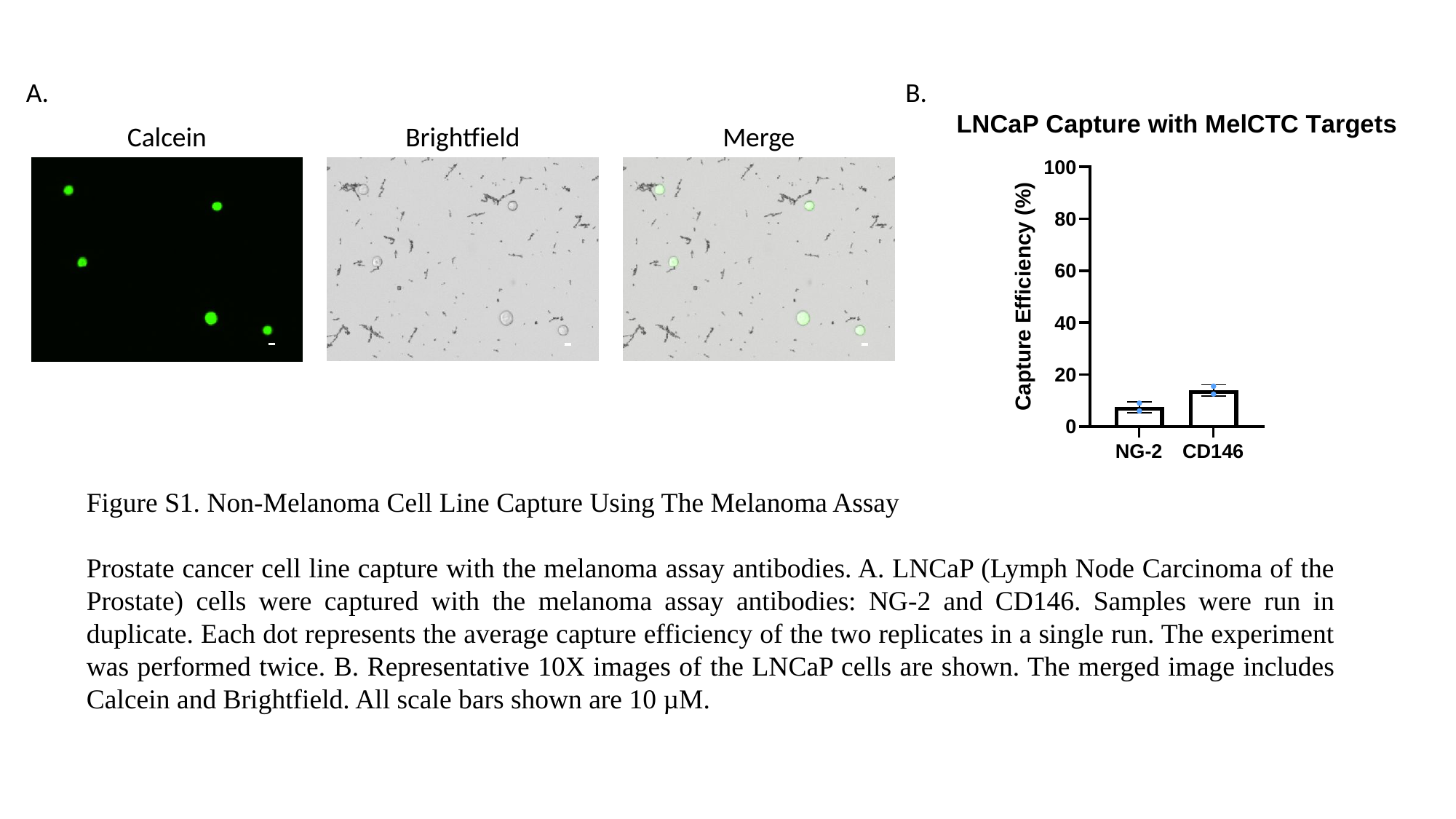

A.
Calcein
Brightfield
Merge
B.
Figure S1. Non-Melanoma Cell Line Capture Using The Melanoma Assay
Prostate cancer cell line capture with the melanoma assay antibodies. A. LNCaP (Lymph Node Carcinoma of the Prostate) cells were captured with the melanoma assay antibodies: NG-2 and CD146. Samples were run in duplicate. Each dot represents the average capture efficiency of the two replicates in a single run. The experiment was performed twice. B. Representative 10X images of the LNCaP cells are shown. The merged image includes Calcein and Brightfield. All scale bars shown are 10 µM.
