# Supplementary material for: Microfluidic Characterization and Analysis of Circulating Tumor Cells From Patients With Metastatic Melanoma
Source: Pigment Cell Melanoma Res. 2025 Jun 2;38(4):e70030. doi: 10.1111/pcmr.70030 (PMC12130673; doi:10.1111/pcmr.70030)
Supplement: Supplementary file 2 — Table S1. Patient characteristics. [file PCMR-38-0-s002.docx]

**Supplementary Table 1:** Patient characteristics

| **Patient Number** | **Age** | **Melanoma Subtype/Origin** | **Disease Stage*** | **Sites of Disease** | **Clinical History** |
| --- | --- | --- | --- | --- | --- |
| Patient 1 | 80s | Cutaneous | IV (M1d) | LN Liver, Spleen. Adrenal Gland, Brain | Multifocal disease with 3+ liver metastases (up to 3.0 cm), splenic metastasis (1.2 cm), unilateral adrenal metastasis (1.9 cm), and 15+ brain metastases (up to 1.2 cm), preceding initiation of ipi/nivo |
| Patient 2 | 70s | Uveal | IV (M1c) | Lung, Pancreas, Liver | Multifocal disease with bilateral lung metastases (up to 1.9 cm), pancreatic metastasis (2.3 cm), and 4+ liver metastases (up to 2.5 cm), preceding initiation of tebe |
| Patient 3 | 70s | Cutaneous | IV (M1d) | LN , Lung, Bone, Peritoneum, Brain | Multifocal disease with large volume peritoneal carcinomatosis, bilateral malignant pleural effusion, retroperitoneal soft tissue metastasis (5.5 cm), multi-station pelvic LN metastasis (up to 3.1 cm), and 3+ CNS metastases (up to 2.1 cm) with interval progressive disease on ipi/nivo |
| Patient 4 | 40s | Cutaneous | IV (M1a) | LN | Metastatic disease with retroperitoneal and pelvic LN involvement (up to 2.7 cm) with interval partial response on ipi/nivo |
| Patient 5 | 60s | Cutaneous | IV (M1b) | LN , Lung | Metastatic disease with bilateral hilar LN metastases (up to 3.6 cm) and bilateral lung metastases (up to 3.2 cm) with interval partial response on nivo/rela |
| Patient 6 | 60s | Mucosal | IV (M1b) | LN, Lung | Multifocal disease with bilateral lung metastases (up to 0.9 cm) and unilateral inguinal LN involvement (1.6 cm) with interval partial response on ipi/nivo |
| Patient 7 | 60s | Uveal | IV (M1c) | Liver | Metastatic disease with 4+ liver metastases (up to 3.2 cm) preceding initiation of tebe |
| Patient 8 | 20s | Unknown | IV (M1c) | LN , Liver, Bone | Multifocal disease with bilateral lung metastases (up to 0.5 cm), 3+ liver metastases (up to 2.4 cm), and pelvic osseous metastasis (2.0 cm) with interval progressive disease on ipi/nivo |
| Patient 9 | 30s | Unknown | IV (M1d) | LN, Lung, Bowel, Liver, Spleen, Brain | Multifocal disease with 3+ brain metastases (up to 2.1 cm), bilateral lung metastases (up to 0.8 cm), liver metastasis (1.1 cm), splenic metastasis (1.5 cm), portacaval LN metastasis (4.0 cm), and intraluminal duodenal metastasis (unmeasurable) with interval partial response to ipi/nivo |
| Patient 10 | 40s | Cutaneous | IV (M1c) | LN , Lung, Liver, Bone | Multifocal disease with unilateral supraclavicular LN (5.2 cm), bilateral lung metastases (up to 0.7 cm), femur osseous metastasis (2.3 cm), 10+ liver metastases (up to 4.9 cm), and upper abdominal LN metastases (up to 2.2 cm) receiving treatment with ipi/nivo |

*Per AJCC 8^th^ edition staging for melanoma

Ipi/Nivo: Ipilimumab/Nivolumab; Nivo/Rela: Nivolumab/Relatlimab; Nivo: Nivolumab; Tebe: Tebentafusp; LN: Lymph node; CNS: Central Nervous System

Supplementary Table 1. Patient Clinical Details

Collection of patient clinical details. The table summarizes the items of each patient’s disease history including sites of metastasis, disease subtype, disease stage, and metastasis history.
